# *Foxn1* Is Dynamically Regulated in Thymic Epithelial Cells during Embryogenesis and at the Onset of Thymic Involution

**DOI:** 10.1371/journal.pone.0151666

**Published:** 2016-03-16

**Authors:** Kathy E. O’Neill, Nicholas Bredenkamp, Christin Tischner, Harsh J. Vaidya, Frances H. Stenhouse, C. Diana Peddie, Craig S. Nowell, Terri Gaskell, C. Clare Blackburn

**Affiliations:** MRC Centre for Regenerative Medicine, Institute for Stem Cell Research, School of Biological Sciences, University of Edinburgh, SCRM Building, 5 Little France Drive, Edinburgh, EH16 4UU, UK; University of São Paulo, BRAZIL

## Abstract

Thymus function requires extensive cross-talk between developing T-cells and the thymic epithelium, which consists of cortical and medullary TEC. The transcription factor FOXN1 is the master regulator of TEC differentiation and function, and declining *Foxn1* expression with age results in stereotypical thymic involution. Understanding of the dynamics of *Foxn1* expression is, however, limited by a lack of single cell resolution data. We have generated a novel reporter of *Foxn1* expression, *Foxn1*^*G*^, to monitor changes in *Foxn1* expression during embryogenesis and involution. Our data reveal that early differentiation and maturation of cortical and medullary TEC coincides with precise sub-lineage-specific regulation of *Foxn1* expression levels. We further show that initiation of thymic involution is associated with reduced cTEC functionality, and proportional expansion of FOXN1-negative TEC in both cortical and medullary sub-lineages. Cortex-specific down-regulation of *Foxn1* between 1 and 3 months of age may therefore be a key driver of the early stages of age-related thymic involution.

## Introduction

The thymus is required for T cell commitment, differentiation and repertoire selection. These functions are mediated by the epithelial component of the thymic stroma, which is composed of cortical (c) and medullary (m) thymic epithelial cells (TECs). cTECs and mTECs are spatially separated and functionally distinct. The early stages of T cell development, from commitment to positive selection, occur in the cortex, while central tolerance—including negative selection and T regulatory cell induction—is imposed in the medulla [1].

The transcription factor FOXN1 is required throughout ontogeny for TEC production and function. Null mutations in *Foxn1* result in the *nude* phenotype, characterised by hairlessness and athymia in mice, rats and humans [2–6]. The *nude* thymic primordium forms normally, but TEC development is blocked at the thymic epithelial progenitor cell (TEPC) stage [7, 8]. FOXN1 is also required to maintain the postnatal thymus [9–11]. Age-related thymic involution, a stereotypical process of age-related degeneration, is associated with a progressive decline in *Foxn1* expression [9, 10, 12]. Overexpression of *Foxn1* in young mice is sufficient to delay involution [10], and TEC-specific up-regulation of *Foxn1* in the fully involuted thymus is sufficient to drive robust regeneration that returns the architecture, gene expression and function of the organ to a pre-involution state [13]. Regulation of *Foxn1* mRNA expression is thus a primary component of the mechanism of age-related thymic involution [13].

Orchestration by FOXN1 of the gene expression program that controls TEC differentiation and function depends on precise control of *Foxn1* expression levels [9, 14]. During development, lineage progression of both TEC sub-lineages requires an increasing amount of FOXN1, such that each successive stage of development occurs at a certain threshold dose [14]. Low-level *Foxn1* expression is permissive for exit from the TEPC state and initiation of TEC differentiation, but insufficient to support acquisition of specialist TEC functions including the attraction of lymphoid progenitors [14]. Maintenance of TEC in the adult thymus is similarly sensitive to FOXN1 level, such that reduction of *Foxn1* expression to 30% of wild-type (WT) levels results in premature involution [9]. Despite its importance in establishing and maintaining functional TEC, understanding of the dynamics of *Foxn1* expression level remains limited due to a lack of single cell data.

To interrogate *Foxn1* expression at single cell resolution, we generated and analyzed a novel transgenic mouse line in which activation of the *Foxn1* promoter is faithfully reported by GFP expression. Our data reveal that *Foxn1* is differentially expressed and dynamically regulated in both the fetal and adult thymus. In particular, initiation of age-related thymic involution occurs concurrently with cTEC-specific down-regulation of *Foxn1* and the rapid proportional expansion of a population of *Foxn1*-negative TEC. Collectively, our data implicate dynamic regulation of *Foxn1* as a requirement for both TEC sub-lineage development and the initiation of age-related thymic involution, and suggest that reduced cTEC functionality resulting from decreased *Foxn1* expression is a primary instigator of the age-associated decline in thymic cellularity and output.

## Materials and Methods

### Animal Research

#### Ethics statement

This study was carried out in strict accordance with UK Home Office guidelines, as established in the ANIMALS (SCIENTIFIC PROCEDURES) ACT 1986. The experimental protocol was approved by the UK Home Office under Project Licence PPL60/4435.

#### Mice

Male C57BL/6 or *Foxn1*^*G/+*^ mice were used for isolation of postnatal TEC. For timed matings, *Foxn1*^*G/+*^ females were housed with CBA males and noon of the day of the vaginal plug was assumed to be E0.5. All animals were housed and bred at University of Edinburgh animal facilities.

#### Genotyping

Mice were genotyped by PCR using the following primers (shown 5' to 3'):

Forward: CAAGTCCTCGTTCAGCATCA; Reverse: GCTTCTCGTACTTGCCGTTC.

### Flow cytometry

**Fig 1:**
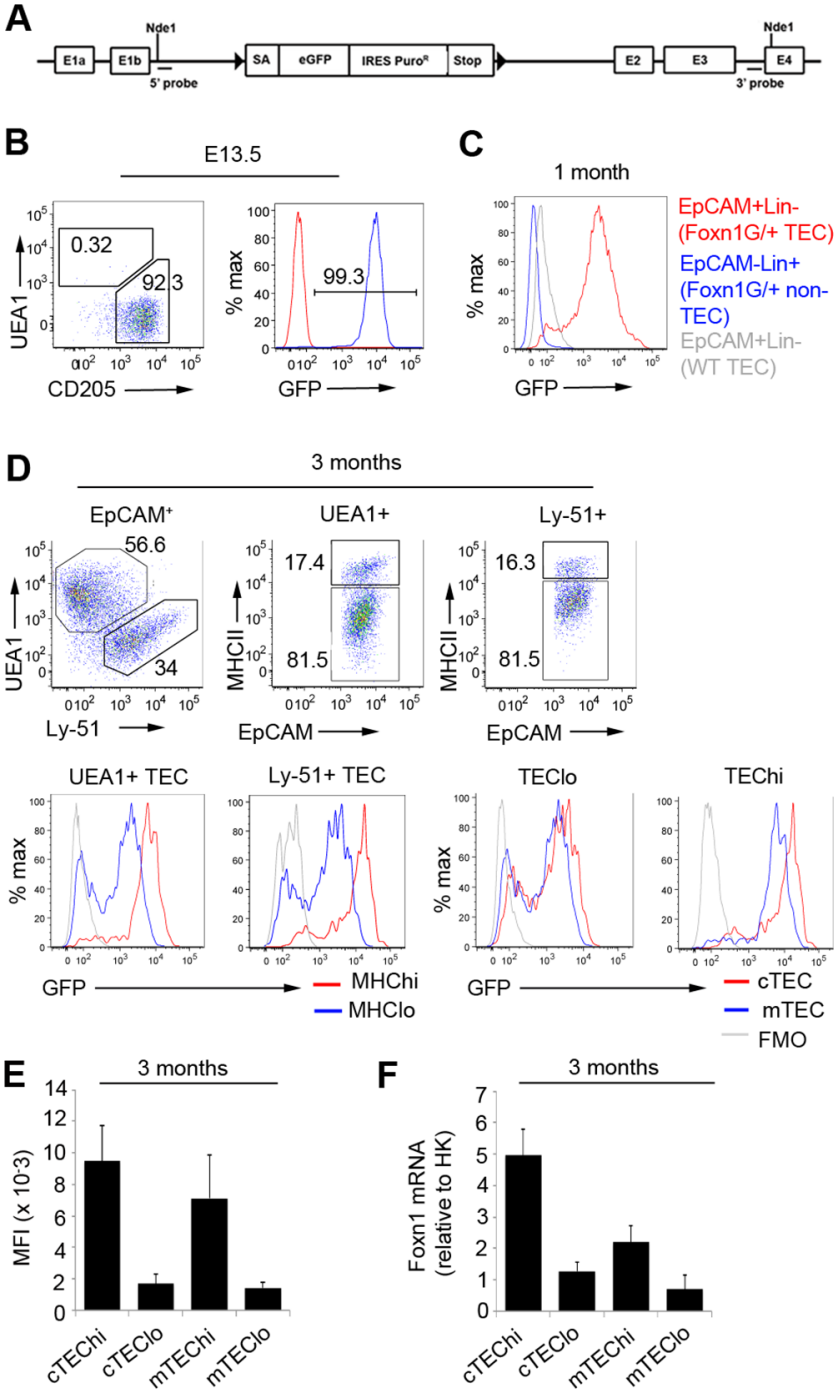
Generation and validation of *Foxn1*^*G*^ reporter mice. **(A)** Schematic representation of the *Foxn1*^*G*^ allele. A LoxP flanked cassette containing the 5’ engrailed 2 splice acceptor site (SA), eGFP, an internal ribosome entry site coupled to the puromycin resistance fusion protein (IRES-Puro^R^), and the CMAZ transcriptional pause (Stop) [19] was inserted into intron 1b of the *Foxn1* locus of mouse ES cells. E, exon. **(B)** Flow cytometric analysis of E13.5 *Foxn1*^*G/+*^ thymic primordia. Plots show data after gating against Lineage^+^ cells (Lin) and on total EpCAM^+^ cells. Left plot shows analysis with UEA1 and anti-CD205. WT, wild type. Red line shows FMO. **(C)** Flow cytometric analysis of thymi from 1 month old *Foxn1*^*G/+*^ or WT mice. Plots display data from total EpCAM^+^Lin^-^ or EpCAM^-^Lin^+^, as shown. Absolute number of EpCAM^+^ cells for 1 month old *Foxn1*^*G/+*^ mice, 6.06x10^4^±2.10 x 10^4^. (**D)** Flow cytometric analysis of 3 month old *Foxn1*^*G/+*^ thymi after staining with the markers shown. Plots show subpopulations of EpCAM^+^ cells, as shown. Absolute number of EpCAM^+^ cells for 3 month old *Foxn1*^*G/+*^ mice, 5.41x10^4^±6.97x10^3^. **(E)** Median fluorescence intensities (MFI) for data shown in **(D). (F)** RT-qPCR analysis showing relative *Foxn1* mRNA expression level in the populations shown, after purification by flow cytometry. **(B)** n = 4, (**C**) n = 5, (**D,E**) n = 6, (**F**) n = 5 independent biological experiments.

Fetal and postnatal thymi were prepared for flow cytometric analysis and sorting as previously described [13–15]. Briefly, postnatal thymi were dissociated in RPMI-1640 HEPES medium (Life Technologies) with 1.25mg/ml collagenase D (Roche) and 0.05mg/ml DNase I (Roche) for 3x15 minutes at 37°C. The remaining tissue was digested in RPMI-1640 HEPES medium with 1.25mg/ml collagenase/dispase (Roche) and 0.05mg/ml DNase I for 30 minutes at 37°C. CD45^+^ cells were depleted using α-CD45 magnetic beads (Miltenyi Biotech) in conjunction with LS columns (Miltenyi Biotech) and a QuadroMACS (Miltenyi Biotech) according to the manufacturer’s instructions. CD45^+^ cells were not depleted for analyses requiring absolute numbers of TEC. Antibodies used in flow cytometry are shown in S1 Table. Isotype controls were included in analyses where appropriate. DAPI was used to discriminate dead cells in experiments not involving intracellular staining. Lineage+ cells (Lineage = CD3, CD4, CD8, Ter119, CD11c, CD31) were excluded (except for Fig 1C). For Ki67 and active caspase-3 analyses, GFP-positive and GFP-negative TEC were isolated by FACS prior to analysis by flow cytometry. Fixable viability dye eFluor450 (eBioscience) was used to identify dead cells. Samples were fixed with BD CytoFix/CytoPerm (BD Biosciences) and permeabilized using BD Perm/Wash (BD Biosciences) according to the manufacturer’s instructions. All flow cytometry data were analyzed using FlowJo Version 9.7.7 (Tree Star, Inc).

### RT-qPCR

100 cells of the required phenotype were sorted by flow cytometry into 10μl CellsDirect 2x Reaction Mix (Life Technologies) and 4U SUPERase In RNase inhibitor (Ambion). cDNA was synthesized and pre-amplified in one step by addition of 1μl CellsDirect SuperScript III reverse transcriptase / Platinum Taq mix (Life Technologies) and target-specific primers at a final concentration of 50nM. Primer sequences are shown in S2 Table. Thermal cycling conditions were as follows: 50°C for 15 minutes; 95°C for 2 minutes; 18 cycles of 95°C for 15 seconds, 60°C for 4 minutes. qPCR was conducted using the Roche Universal Probe Library (Roche) and LightCycler 480 real-time PCR instrument (Roche) according to the manufacturer’s instructions. Amplification conditions were as follows: 95°C for 5 minutes; 40 cycles of 95°C for 10 seconds, 60°C for 20 seconds. Data were normalised to the geometric mean of three housekeepers (*Hprt*, *Ywhaz*, *Hmbs*). All samples were run in triplicate and no RT and no template controls were included in all experiments. Data were analyzed using LightCycler 1.5 software (Roche) and the ΔCt method.

### Statistical analysis

Statistical analysis was performed using Student’s T-test. p-values <0.05 were considered significant. Errors shown are standard deviations throughout. Sample sizes of at least n = 3 independent biological experiments were used for statistical analyses. For adult analyses, n is a single mouse or a single group of mice. For fetal analyses, n is a pool of genetically identical TEC isolated from a single litter.

## Results

### Generation and validation of a *Foxn1-eGFP* reporter mouse line

To determine *Foxn1* expression levels in individual TEC, we generated a transgenic mouse line in which a LoxP site-flanked cassette containing the *eGFP* cDNA, an IRES element, the cDNA encoding puromycin resistance and a transcriptional stop sequence, was knocked into intron 1b of the *Foxn1* locus (NCBI Gene ID: 15218) by homologous recombination in ES cells generating a revertible functionally null allele, *Foxn1*^*G*^ (Fig 1A and S1 Fig). Preliminary analysis demonstrated GFP expression in the fetal thymus and adult hair shafts of *Foxn1*^*G/+*^ and *Foxn1*^*G/G*^ mice (not shown). To test fidelity of reporter expression, we analyzed fetal TEC at day 13.5 of embryonic development (E13.5). At this stage, 99.6±0.3% of EpCAM^+^ epithelial cells were GFP^+^ (Fig 1B), consistent with earlier reporter and expression analyses [16, 17] and with the finding that all TEC in the adult thymus are derived from a *Foxn1*^*+*^ lineage [11]. As expected, GFP was not expressed in T-cells or endothelial cells at any developmental stage (shown for 1 month old in Fig 1C). We next compared *Foxn1* mRNA and eGFP levels in four subsets of postnatal TEC: cTEC MHC Class II^hi^ (cTEC^hi^), cTEC MHC Class II^lo^ (cTEC^lo^), mTEC MHC Class II^hi^ (mTEC^hi^) and mTEC MHC Class II^lo^ (mTEC^lo^), defined using the cell-surface markers UEA1 (mTEC), Ly-51 (cTEC) and MHC Class II (MHCII). As previously described [14, 18], MHCII^hi^ TEC expressed more *Foxn1* mRNA than MHCII^lo^ TEC in both cortex and medulla, and cTEC expressed *Foxn1* more highly than mTEC of equivalent MHCII status (Fig 1D and 1E). GFP expression in each TEC subset correlated closely with *Foxn1* mRNA levels (Fig 1E and 1F). These data validated the *Foxn1*^*G/+*^ reporter line and demonstrated its capacity to reliably detect differences in *Foxn1* expression of as little as 1.7-fold (Fig 1F).

### Heterogeneity of *Foxn1* expression is established during fetal development

**Fig 2:**
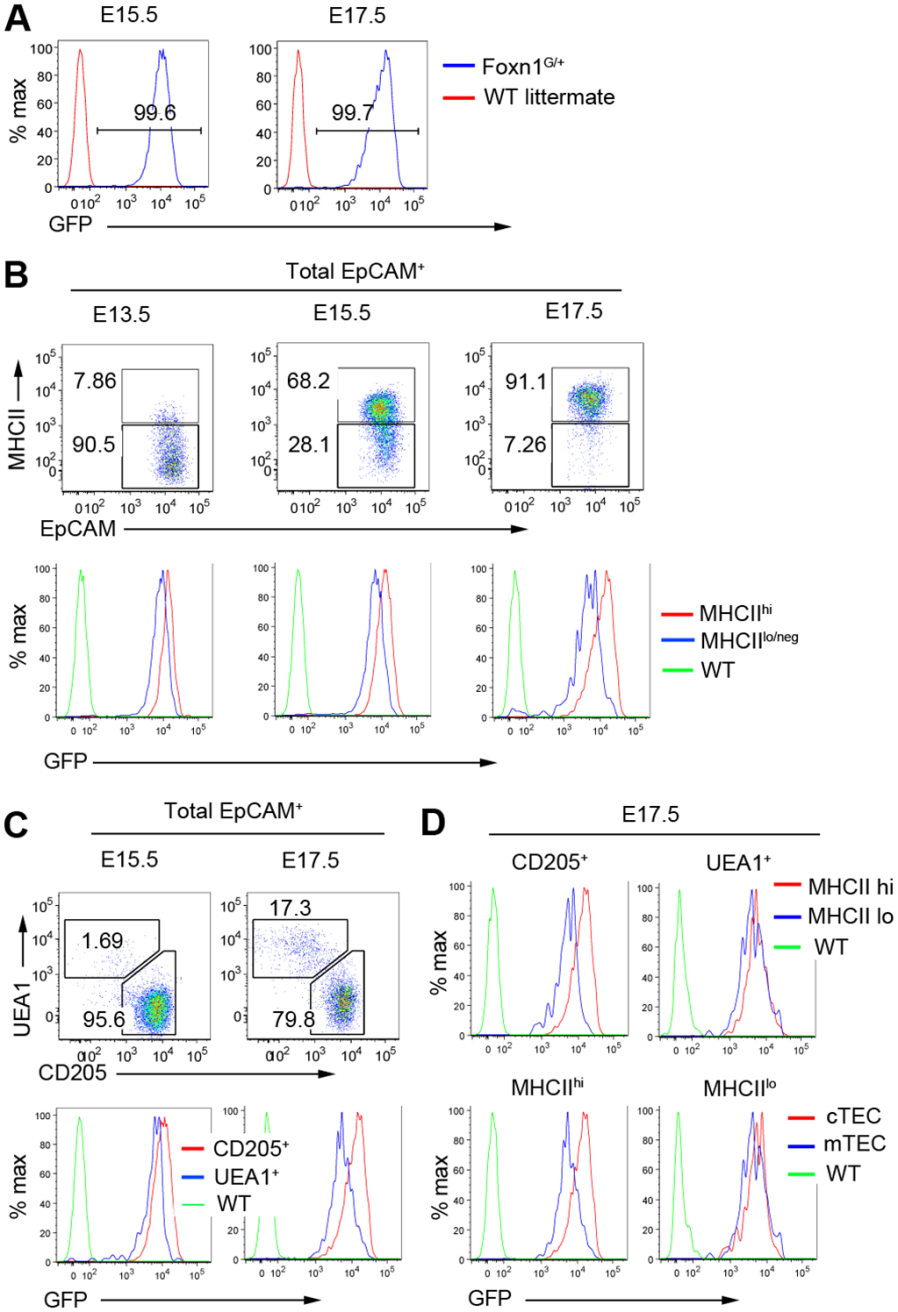
Dynamic regulation of *Foxn1* is evident in the early stages of TEC differentiation. **(A-D)** Flow cytometric analysis of E13.5, E15.5 and E17.5 fetal *Foxn1*^*G/+*^ thymic primordia for the markers shown. Plots show data after gating against Lineage^+^ and on total EpCAM^+^ cells. WT, wild type. **(A-C)** n = 3, (**D**) n = 4 independent biological experiments.

To investigate the origin of the differential *Foxn1* expression characteristic of different postnatal TEC sub-lineages, *Foxn1*^*G/+*^ thymi were analyzed at E13.5, E15.5 and E17.5 (Figs 1B and 2). By E13.5, the thymus and parathyroid domains of the common primordium have resolved into two discrete organs, preventing contamination of TEC analyses by *Foxn1*-negative parathyroid cells. At each stage, more than 99% of EpCAM^+^ TEC were GFP^+^ (Figs 1B and 2A, [20]) and MHCII^hi^ cells expressed more GFP than MHCII^lo^ cells (Fig 2B). At E17.5, when an appreciable UEA1^+^ population is first apparent in *Foxn1*^*G/+*^ embryos, GFP expression was higher in CD205^+^ cTEC than in UEA1^+^ mTEC with equivalent MHCII levels (Fig 2C and 2D). Downregulation of *Foxn1* expression in *Foxn1*^hi^ TEPC may therefore be required for mTEC divergence or expansion, or alternatively, UEA1^+^ TEC may be derived from a *Foxn1*^lo^ lineage. In sum, the graded expression of *Foxn1* observed in the adult thymic epithelium is present from the earliest stages of TEC differentiation, linking *Foxn1* dosage to the early differentiation and maturation of cortical and medullary TEC.

### *Foxn1* negative TEC in the postnatal thymus

**Fig 3:**
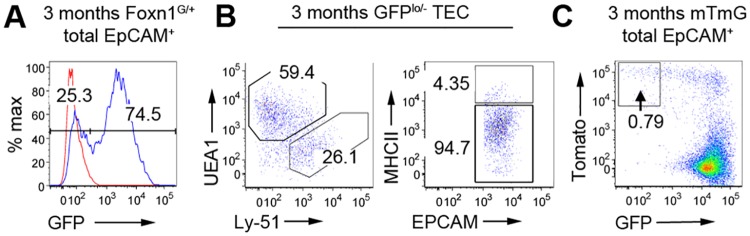
*Foxn1*^*neg*^ TEC subpopulations emerge postnatally in both cTEC and mTEC compartments. **(A-C)** Flow cytometric analysis of thymi from 12 week-old adult *Foxn1*^*G/+*^ (**A,B)** or *Foxn1*^*Cre*^*;mTmG* (**C**) mice, for the markers shown. Data are shown after gating against Lineage^+^ and on total EpCAM^+^ cells **(A-C)** and further gating on GFP^neg^ cells **(B)**. Absolute number of GFP^neg^ cells in 3 month old mice, 1.31x10^4^±3.01x10^3^. WT, wild type. Red line in (**A**) shows FMO. **(A,B)** n = 6, (**C**) n = 3 independent biological experiments.

The above analyses revealed a GFP-negative subpopulation in postnatal *Foxn1*^*G/+*^ TEC, representing 24.8±7.6% of total TEC in 3 month-old mice (Fig 3A). These *Foxn1*-negative cells were present in the MHCII^hi^ and MHCII^lo^ fractions of both cTEC and mTEC, but were predominantly MHCII^lo^ (90.7±3.1% of total GFP-negative cells were MHCII^lo^) (Fig 3B). Downregulation of *Foxn1* is a primary component of age-related thymic involution [13], and the presence of *Foxn1-*negative postnatal thymic epithelial stem cell (TESC), derived from a *Foxn1*-negative lineage has also been suggested [21]. The GFP-negative TEC observed in *Foxn1*^*G/+*^ mice may therefore result from down-regulation of *Foxn*1 in *Foxn1*-positive TEC or expansion of a rare *Foxn1*-negative TEC population (>99% of EpCAM^+^ TEC are GFP (*Foxn1*)^+^ throughout fetal thymic development, Figs 1B and 2A). Previous lineage tracing of *Foxn1*-negative TEC using a Foxn1::Cre mouse line suggested that these cells arise from *Foxn1*-positive precursors [11]. However, Cre was driven from a *Foxn1* promoter fragment, which may not precisely recapitulate endogenous *Foxn1* expression. Therefore, to test the origins of the postnatal GFP*(Foxn1)*-negative TEC identified here, *Foxn1*^*Cre*^ mice were crossed with *Gt(ROSA)26*^*Sortm4(ACTB-tdTomato*,*-EGFP)Luo/J*^ reporter mice (*mT*/*mG* mice) [22] in which tdTomato expression is extinguished and eGFP expression activated upon Cre-mediated recombination of the reporter locus. Of TEC isolated from 9 week to 3 month-old *Foxn1*^*Cre/+*^*;mT/mG* mice, 0.5±0.3% were GFP-negative (shown for 3 month-old in Fig 3C). In comparison, 24.8±7.6% of TEC in 3 month-old *Foxn1*^*G/+*^mice are GFP negative (Fig 3A). These data establish that expansion of a minor TEC subpopulation that never expresses *Foxn1* cannot account for the GFP (*Foxn1)*-negative TEC population present in the adult *Foxn1*^*G/+*^ thymus: if this was the case, the proportion of GFP^-^ cells in the *Foxn1*^*Cre/+*^*;mT/mG* reporter mice and *Foxn1*^*G/+*^ mice would be equivalent. Thus, the GFP-negative TEC in the adult *Foxn1*^*G/+*^ thymus must be generated by down-regulation of *Foxn1* in previously *Foxn1-*positive TEC.

### Proportional expansion of *Foxn1* negative TEC is associated with thymic involution

**Fig 4:**
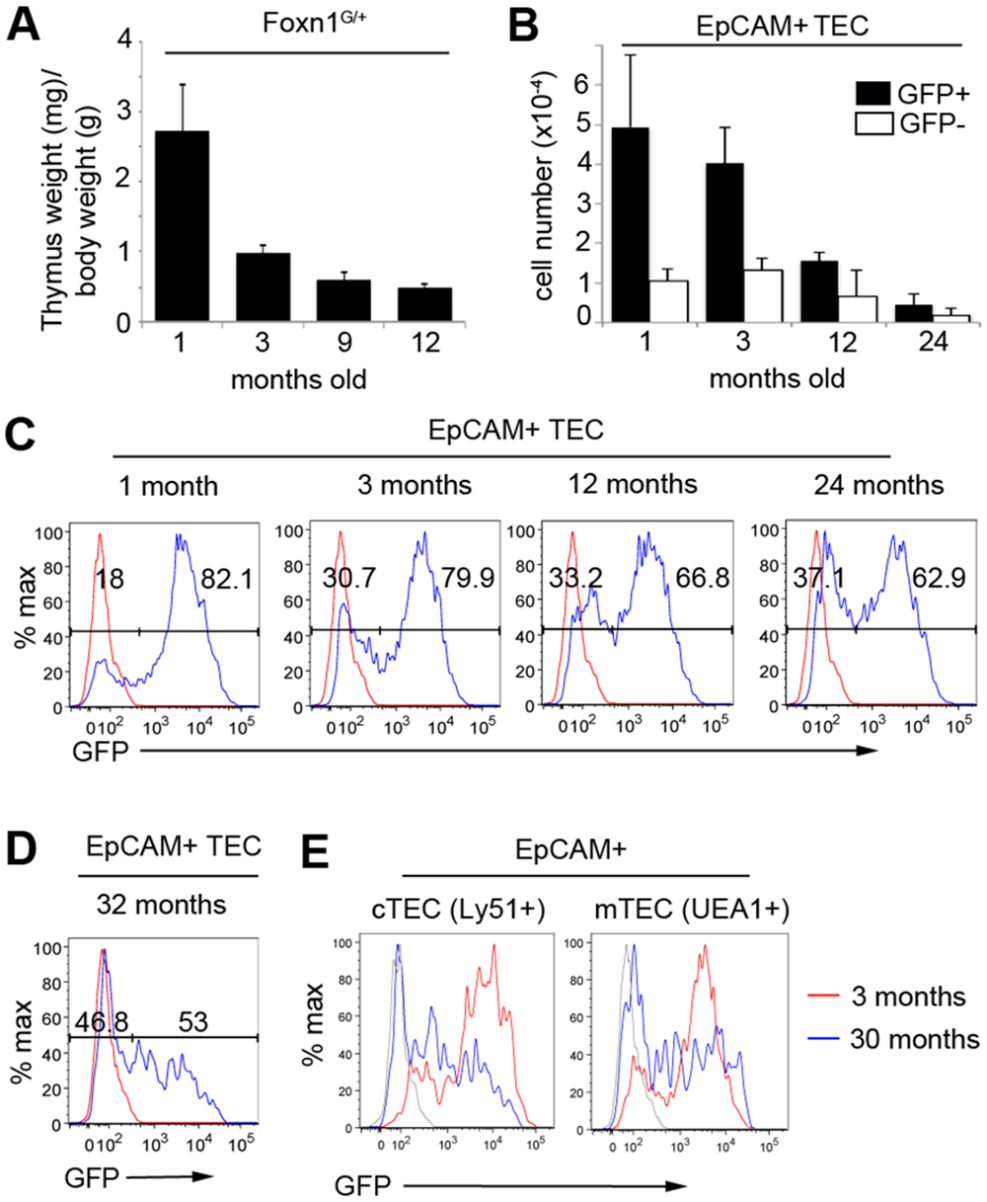
Proportional expansion of *Foxn1*^*neg*^ TEC occurs at the onset of age-related thymic involution. **(A)** Thymus involution in *Foxn1*^*G/+*^ mice occurs with normal kinetics. **(C, D, E)** Flow cytometric analysis of *Foxn1*^*G/+*^ thymi at the ages shown; the proportion of GFP- TEC increases with age. Red (C, D) and grey (E) lines show FMO. (**B**) Absolute number of GFP^+^ and GFP^-^ TEC isolated from *Foxn1*^*G/+*^ thymi at the timepoints shown. Absolute numbers are as follows: 1 month; GFP^+^ 4.94x10^4^±1.81x10^4^, GFP^-^ 1.06x10^4^±3.07x10^3^. 3 months; GFP^+^ 4.03x10^4^±8.86x10^3^, GFP^-^ 1.31x10^4^±3.01 x 10^3^. 12 months; GFP^+^ 1.55x10^4^±2.36x10^3^, GFP^-^ 6.55x10^3^±1.88x10^3^. 24 months; GFP^+^ 4.52x10^3^±2.75x10^3^, GFP^-^ 1.81x10^3^±1.10x10^3^. **(A,B,C)** n = 3, (**D,E**) n = 2 independent biological experiments.

Down-regulation of *Foxn1* has been implicated as a cause of thymic involution [9, 10, 12, 13], but the dynamics of *Foxn1* expression during ageing have not been reported at the single cell level in defined TEC subsets. Age-related decline in thymus weight and cellularity occurred with the same kinetics in *Foxn1*^*G/+*^ as in WT mice (Fig 4A). Therefore, to investigate the relationship between the *Foxn1*-negative TEC population and postnatal thymus function, we interrogated GFP (*Foxn1*) expression at a range of ages spanning the initiation and progression of thymic involution. The absolute number of both GFP^+^ and GFP^-^ TEC decreased in *Foxn1*^*G/+*^ mice between 1 month and 2 years old (p = 0.0007, p = 0.0003 for GFP^+^ and GFP^-^ TEC respectively) (Fig 4B). However the proportion of GFP-negative TEC increased; at 1 month, 3 months, 12 months and 24 months old, 18.0±4.1%, 24.8±7.6%, 28.7±3.2% and 29.3±7.9% of TEC were GFP-negative respectively (p = 0.05, p = 0.43, p = 0.90 for 1 month v 3 months, 3 months v 1 year, 1 year v 2 years respectively)(Fig 4C). Thus, the proportion of GFP-negative TEC increases rapidly between 1 month and 3 months postnatal and is stable thereafter, consistent with a recent report based on antibody staining [20]. These two phases correlate inversely with the initially fast and then slower decrease in thymus size characteristic of the onset and progression of thymic involution (Fig 4A), suggesting that involution is a consequence of a proportional increase in subfunctional, Foxn1-negative TEC. However, our data demonstrate that age-related down-regulation of *Foxn1* is not uniform in all TEC; some TEC continue to express high *Foxn1* (GFP) levels even in very old mice (Fig 4D and 4E).

### Initiation of thymic involution correlates with decreased *Foxn1* expression in cTEC

**Fig 5:**
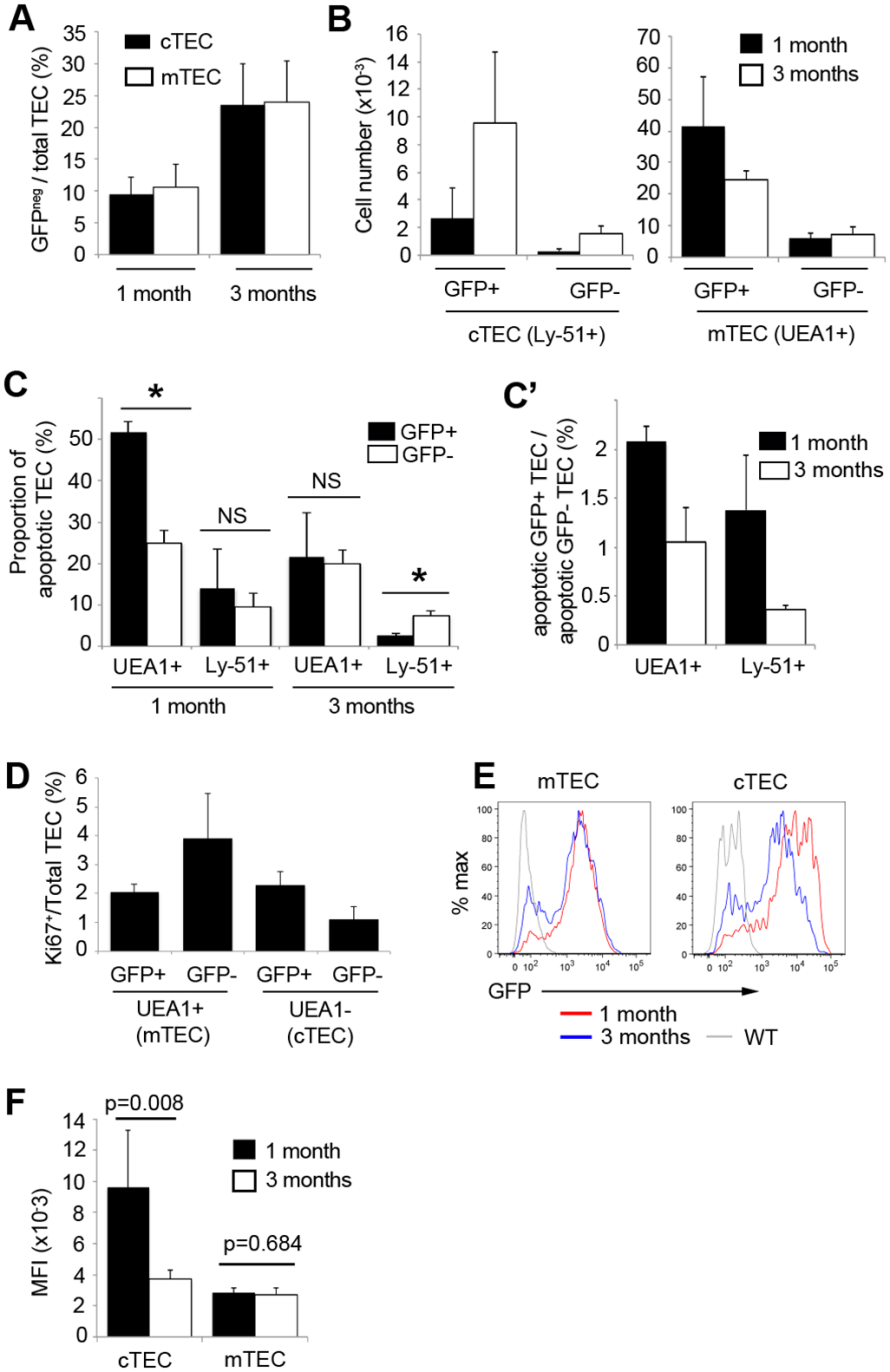
Proportional expansion of *Foxn1*^*neg*^ cTEC and mTEC is driven by different mechanisms. **(A,B)** Plots show proportion of GFP^-^ **(A)** and numbers of GFP^+^ and GFP^-^ (B) mTEC and cTEC at the ages shown, as determined by flow cytometric analysis. (**C**) Flow cytometric analysis showing proportion of active caspase-3^+^ cells in the populations shown. (**C’**) shows data in (**C**) presented to indicate the relative levels of apoptosis in GFP^+^ and GFP^-^ cTEC and mTEC. **(D)** Flow cytometric analysis of thymi from 6 week old mice showing proportion of Ki67^+^ cells in the populations shown. **(E)** GFP expression profile in cTEC and mTEC at the ages shown. (F) Quantification of data displayed in (E) showing median fluorescence intensity (MFI) of GFP^+^ cTEC and mTEC at 1 month and 3 months. **(A)** n = 5, (B) n = 7, (**C**) n = 3 (**D**) n = 4, (**E, F**) n = 5 independent biological experiments.

The rapid proportional expansion of GFP-negative TEC between 1 month and 3 months of age suggested a link between this process and the initiation of age-related thymic involution. We therefore investigated whether age-associated changes in GFP expression occurred with the same kinetics in cTEC and mTEC. The proportion of GFP-negative TEC was equivalent in the cortex and medulla at 1 month and 3 months, and increased in both subsets between 1 and 3 months of age (p = 0.0031, p = 0.0004 for cTEC and mTEC respectively) (Fig 5A). In mTEC, this proportional change reflected a significantly decreased number of GFP^+^ mTEC in 3- compared to 1-month old mice (4.13x10^4^±1.59x10^4^ and 2.47x10^4^±2.77x10^3^ GFP^+^ mTEC at 1 month and 3 months respectively, p = 0.018). In cTEC, both GFP^+^ and GFP^-^ cells increased numerically between 1 and 3 months of age (p = 0.004, p = 0.00001 respectively) (Fig 5B).

To investigate how thymic epithelial composition is altered during early involution, we analysed cell death in GFP+ and GFP- cTEC and mTEC at 1 month and 3 months, and also determined proliferation rate in the same TEC subsets. At 1 month, a higher proportion of GFP^+^ than GFP^-^ mTEC was apoptotic (51.7%±2.5% and 25.0%±3.0% of GFP^+^ and GFP^-^ mTEC respectively, p = 0.0003), whereas at 3 months the frequency of apoptosis was equivalent in GFP^+^ and GFP^-^ mTEC (21.6%±10.6% and 19.9%±3.3% of GFP^+^ and GFP^-^ mTEC respectively, p = 0.803) (Fig 5C and 5C’). Additionally, GFP^+^ mTEC were less proliferative than GFP^-^ mTEC in 6 week-old mice (p = 0.05) (Fig 5D). These data indicate that apoptosis of GFP^+^ mTEC contributes to the proportional expansion of GFP^-^ mTEC between 1 and 3 months of age, and suggest that a difference in proliferative index may also contribute to the increased proportion of GFP^-^ mTEC at 3 months old.

In cTEC, the proportion of apoptotic cells was not significantly different in the GFP^+^ and GFP^-^ subsets at 1 month (13.9%±9.6% and 9.6%±3.1% of GFP^+^ and GFP^-^ cTEC respectively, p = 0.50), but was increased in GFP^-^ compared to GFP^+^ cTEC at 3 months (2.65%±0.6% and 7.31%±1.2% of GFP^+^ and GFP^-^ cTEC respectively, p = 0.004). Additionally, GFP^+^ cTEC were more proliferative than GFP^-^ cTEC at 6 weeks old (p = 0.009, Fig 5D). Thus in contrast to mTEC, the increased proportion of GFP^-^ cTEC in 3 month old mice cannot be explained by apoptosis of GFP^+^ cells and differences in proliferation rate between GFP^+^ and GFP^-^ TEC. Further analysis indicated that the median level of GFP expression in GFP^+^ cTEC declines between 1 and 3 months (p = 0.008, Fig 5E and 5F). These data suggest that the accumulation of GFP^-^ cTEC in 3 month old mice primarily results from specific downregulation of *Foxn1* in a GFP^+^ cTEC subpopulation that subsequently persists in the epithelium. This broad *Foxn1* down-regulation was not observed in GFP^+^ mTEC. Thus, our data collectively indicate that the earliest stages of involution are driven by different mechanisms in the cortex and medulla.

### Down-regulation of *Foxn1* results in loss of cTEC function at the onset of involution

**Fig 6:**
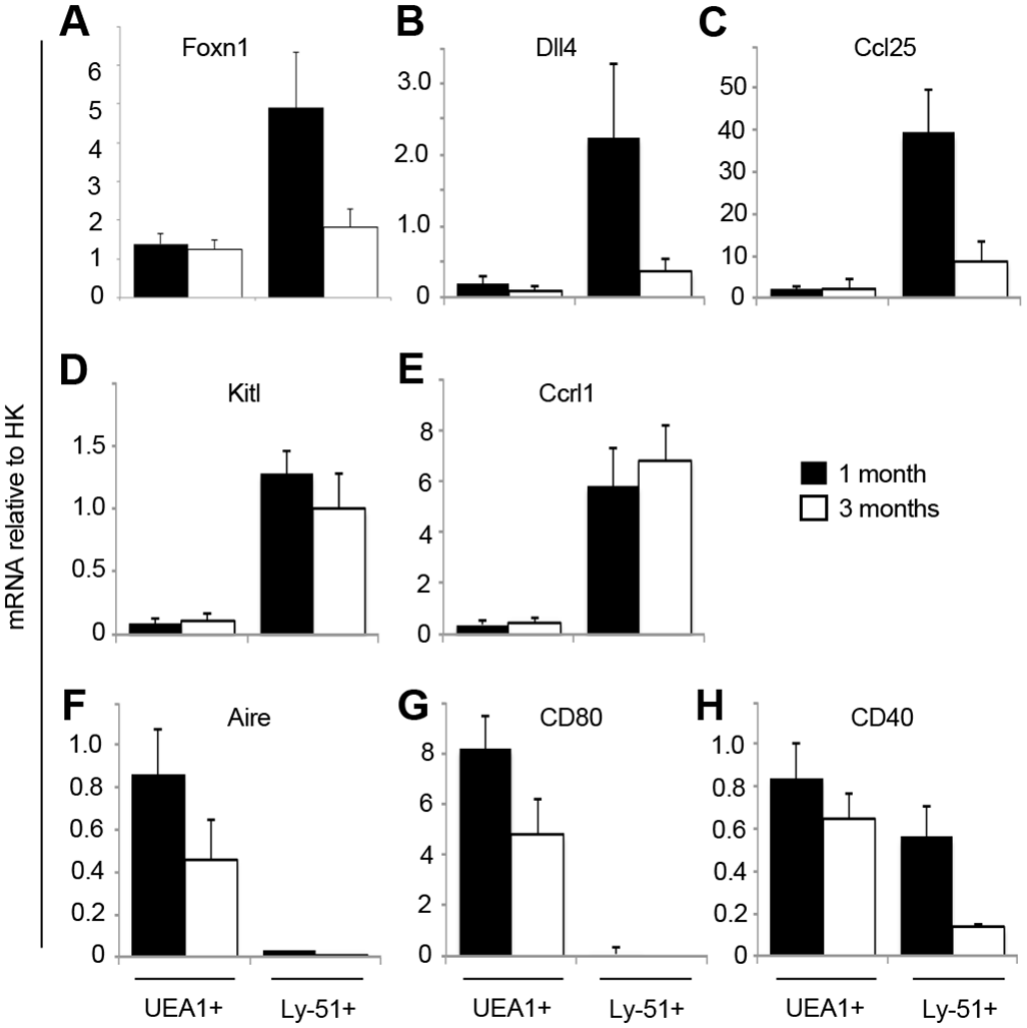
Changes in gene expression with age. **(A-H)** Graphs show RT-qPCR analysis of the markers shown in WT mice at 1 month and 3 months. Error bars show SD. (**A-G**) n = 5, (**H**) n = 3 independent biological experiments.

In keeping with the observed decrease in median GFP expression levels in GFP^+^ cTEC, RT-qPCR analysis of WT mice showed significantly reduced *Foxn1* mRNA levels in cTEC (*p* = 0.008) but not in mTEC (*p =* 0.485) between 1 and 3 months of age (Fig 6A). FOXN1 in cTEC regulates the expression of several factors required for attraction, specification and proliferation of thymocytes, including *Dll4*, *Ccl25* and *KitL* [13, 14]. RT-qPCR analysis showed a significant reduction in expression of *Dll4* and *Ccl25* in WT cTEC between 1 and 3 months old (*p* = 0.013 and *p* = 0.001 respectively)(Fig 6B and 6C). *KitL* and *Ccrl1*, in contrast, were not significantly down-regulated (*p* = 0.224 and *p* = 0.362 respectively)(Fig 6D and 6E). In the medulla, *Foxn1* expression was maintained between 1 and 3 months of age but the expression of *Aire* and *Cd80* decreased (*p* = 0.029 and *p* = 0.017 respectively)(Fig 6F and 6G). *Cd40* was significantly down-regulated in cTEC (*p* = 0.041), but not in mTEC (*p* = 0.128)(Fig 6H). Thus cortex-specific down-regulation of *Foxn1* between 1 and 3 months of age may be a key driver of the early stages of involution.

## Discussion

Using the *Foxn1*^*G*^ reporter mouse, we have conducted a single cell resolution analysis of the level and dynamics of *Foxn1* expression in defined subsets of fetal and postnatal TEC. Our data reveal that early fetal mTEC express lower levels of *Foxn1* than their cTEC counterparts, suggesting either that downregulation of *Foxn1* expression in FOXN1-high TEPC is required for mTEC divergence or expansion, or that a *Foxn1*-lo mTEC progenitor is present from the earliest stages of thymus organogenesis. We further show that the onset of thymic involution correlates with rapid proportional expansion of *Foxn1*-negative TEC in both TEC sublineages, and broad down-regulation of *Foxn1* in cTEC. Finally, we show that c-TEC specific downregulation of *Foxn1* is associated with reduced expression of genes required to promote thymocyte commitment, proliferation and differentiation, suggesting that age-related thymic involution is initiated by reduced cTEC function.

### Lineage progression of postnatal mTEC correlates with regulation of *Foxn1* expression

The lineage relationships between the different subpopulations of postnatal mTEC have not yet been clearly defined *in vivo*. However adult mTEC MHCII^lo^ can give rise to mTEC MHCII^hi^ when combined with single positive thymocytes in reaggregate thymic organ culture [23]. Similarly, 70% of postnatal Cldn3/4^hi^ SSEA1^+^ TEC, which have some mTEC progenitor activity, are MHCII^lo/neg^ in 4 week old mice [24]. Our single cell resolution data show that almost all MHCII^lo^ mTEC express less *Foxn1* than MHCII^hi^ mTEC, and therefore indicate that the transition from MHCII^lo^ mTEC to MHCII^hi^ mTEC is associated with up-regulation of *Foxn1*. Recently, it was also shown that MHCII^lo^ cTEC can give rise to both cTEC and mTEC in ectopically grafted reaggregates. Consistent with mTEC^lo^ being precursors of mTEC^hi^ TEC, UEA1^+^ cells appeared in these grafts before MHCII was up-regulated in either sub-lineage [25]. Our data show that, per cell, *Foxn1* is expressed at an equivalent or higher level in Ly-51^+^MHCII^lo^ TEC compared to UEA1^+^MHCII^lo^ TEC, demonstrating that differentiation of UEA1-positive cells from their UEA1-negative precursors does not require up-regulation of *Foxn1*. This observation is consistent with the *Foxn1*-independent divergence of the mTEC lineage in embryogenesis [14], and demonstrates that lineage progression of postnatal mTEC proceeds via two distinct stages, which are respectively independent of and dependent on up-regulation of *Foxn1* expression.

AIRE^+^ mTEC in the postnatal thymus are MHCII^hi^ and CD80^hi^ and are usually considered to be terminally differentiated [23]. However recent lineage tracing has revealed the existence of a post-AIRE population in the thymic medulla [26]. These cells previously expressed AIRE and maintain intermediate levels of tissue specific antigen (TSA) expression, but are MHCII^lo^ and CD80^lo^ [26, 27]. Involucrin, a marker of terminally differentiated epidermis, is enriched in post-AIRE mTEC, and Hassall’s corpuscles contain cells that previously expressed AIRE [27]. Post-AIRE TEC are therefore thought to be a distinct stage of terminal mTEC development. However, our data suggest an alternative possibility, that at least some of these cells are sub-functional TEC produced as a result of involution-associated down-regulation of *Foxn1* and MHCII.

### Thymic involution is initiated in cTEC

The total cellularity of the postnatal thymus is dependent on the number and function of TEC [14, 28]. TEC functionality is particularly important, as increased epithelial proliferation cannot fully compensate for sub-optimal gene expression [14]. Our analyses show that at the onset of involution, down-regulation of *Foxn1* occurs more broadly in cTEC than in mTEC. cTEC-specific targets of FOXN1 include *Ccl25* and *Dll4*, which are required for attraction and specification of thymocytes respectively. Reduced expression of FOXN1 targets in cTEC may thus be the primary driver of declining total cellularity at the initiation of involution. This is consistent with the observation that cortical thinning occurs early in involution and precedes CMJ disorganisation and altered mTEC:cTEC ratio when *Foxn1* is downregulated postnatally [9]. Identification of factors able to modulate FOXN1 expression or function specifically in cTEC could thus provide a novel approach to preventing or reversing thymic involution.

### Rescue of *Foxn1* negative cTEC may facilitate thymus regeneration

The size of the involuted thymus can be at least transiently restored by several approaches, including sex steroid ablation (SSA) and up-regulation of FOXN1. SSA results in thymic rebound through relieving the direct repression of *Dll4* by androgen receptor (AR). Although *Dll4* is expressed in both cTEC and endothelial cells, AR represses *Dll4* specifically in cTEC [29]. SSA-mediated rebound therefore occurs, at least in part, by modulating cTEC activity. cTEC function may also be an important feature of FOXN1-mediated thymic regeneration. When FOXN1 activity is up-regulated in the thymic epithelium of old mice, all TEC populations (cTEC MHC^hi^, cTEC MHC^lo^, mTEC MHC^hi^, mTEC MHC^lo^) expand. However, there is no increase in proliferation of cTEC MHCII^hi^ cells. One possible source of new MHCII^hi^ cTEC is a postnatal progenitor, either bipotent or restricted to the cTEC lineage. Alternatively, some new cTEC MHCII^hi^ could be derived from cTEC that have down-regulated *Foxn1* and MHCII. These cells accumulate in the thymic epithelium as involution progresses, suggesting that functional rescue of *Foxn1*-negative TEC may contribute to thymic rebound. Thus, competence to restore optimal gene expression to sub-functional cTEC may partly explain both the notable regenerative capacity of the thymus and the transient nature of some thymus-regenerating interventions.

Taken together, our single cell-level analyses show that *Foxn1* is tightly and differentially regulated at two stages of thymus ontogeny: first as FOXN1-low mTEC emerge during early thymus organogenesis; and second at the onset of age-related involution. These data suggest that precise manipulation of *Foxn1* expression levels will be necessary for both directed differentiation of the TEC sub-lineages, and sustained endogenous regeneration of the thymus.

## Supporting Information

S1 Fig Characterization of the *Foxn1*^*GFP*^ allele. (PDF)

S1 Table Antibodies used for flow cytometry (PDF)

S2 Table Primers used for RT-qPCR (PDF)

## References

[1] Manley NR, Richie ER, Blackburn CC, Condie BG, Sage J. Structure and function of the thymic microenvironment. Front Biosci (Landmark Ed). 2011;16:2461–77. Epub 2011/05/31. . 21622189 10.2741/3866

[2] Flanagan SP. 'Nude', a new hairless gene with pleiotropic effects in the mouse. Genet Res. 1966;8:295 5980117 10.1017/s0016672300010168

[3] Nehls M, Pfeifer D, Schorpp M, Hedrich H, Boehm T. New member of the winged-helix protein family disrupted in mouse and rat nude mutations. Nature. 1994;372:103–6. 7969402 10.1038/372103a0

[4] Nehls M, Kyewski B, Messerle M, Waldschutz R, Schuddekopf K, Smith AJ, et al Two genetically separable steps in the differentiation of thymic epithelium. Science. 1996;272(5263):886–9. Epub 1996/05/10. . 8629026 10.1126/science.272.5263.886

[5] Frank J, Pignata C, Panteleyev AA, Prowse DM, Baden H, Weiner L, et al Exposing the human nude phenotype [letter]. Nature. 1999;398(6727):473–4. 10206641 10.1038/18997

[6] Jones B, Jesson MI. On the nature of the mutation in the nude rat [letter]. Trends Genetics 1995;11(7):257–8. 10.1016/s0168-9525(00)89068-x 7482772

[7] Blackburn CC, Augustine CL, Li R, Harvey RP, Malin MA, Boyd RL, et al The nu gene acts cell-autonomously and is required for differentiation of thymic epithelial progenitors. Proc Natl Acad Sci USA. 1996;93(12):5742–6. 8650163 10.1073/pnas.93.12.5742 PMC39131

[8] Bleul CC, Corbeaux T, Reuter A, Fisch P, Monting JS, Boehm T. Formation of a functional thymus initiated by a postnatal epithelial progenitor cell. Nature. 2006;441(7096):992–6. . 16791198 10.1038/nature04850

[9] Chen L, Xiao S, Manley NR. Foxn1 is required to maintain the postnatal thymic microenvironment in a dosage-sensitive manner. Blood. 2009;113(3):567–74. 10.1182/blood-2008-05-156265 18978204 PMC2628364

[10] Zook EC, Krishack PA, Zhang S, Zeleznik-Le NJ, Firulli AB, Witte PL, et al Overexpression of Foxn1 attenuates age-associated thymic involution and prevents the expansion of peripheral CD4 memory T cells. Blood. 2011;118(22):5723–31. Epub 2011/09/13. 10.1182/blood-2011-03-342097 21908422 PMC3228493

[11] Corbeaux T, Hess I, Swann JB, Kanzler B, Haas-Assenbaum A, Boehm T. Thymopoiesis in mice depends on a Foxn1-positive thymic epithelial cell lineage. Proc Natl Acad Sci U S A. 2010;107(38):16613–8. Epub 2010/09/09. 10.1073/pnas.1004623107 20823228 PMC2944754

[12] Ortman CL, Dittmar KA, Witte PL, Le PT. Molecular characterization of the mouse involuted thymus: aberrations in expression of transcription regulators in thymocyte and epithelial compartments. Int Immunol. 2002;14(7):813–22. . 12096041 10.1093/intimm/dxf042

[13] Bredenkamp N, Nowell CS, Blackburn CC. Regeneration of the aged thymus by a single transcription factor. Development. 2014;141(8):1627–37. Epub 2014/04/10. 10.1242/dev.103614 . 24715454 PMC3978836

[14] Nowell CS, Bredenkamp N, Tetelin S, Jin X, Tischner C, Vaidya H, et al Foxn1 regulates lineage progression in cortical and medullary thymic epithelial cells but is dispensable for medullary sublineage divergence. PLoS Genet. 2011;7(11):e1002348 Epub 2011/11/11. 10.1371/journal.pgen.1002348 22072979 PMC3207875

[15] Bennett AR, Farley A, Blair NF, Gordon J, Sharp L, Blackburn CC. Identification and characterization of thymic epithelial progenitor cells. Immunity. 2002;16(6):803–14. . 12121662 10.1016/s1074-7613(02)00321-7

[16] Gordon J, Bennett AR, Blackburn CC, Manley NR. Gcm2 and Foxn1 mark early parathyroid- and thymus-specific domains in the developing third pharyngeal pouch. Mech Development. 2001;103(1–2):141–3. . 11335122 10.1016/s0925-4773(01)00333-1

[17] Itoi M, Tsukamoto N, Amagai T. Expression of Dll4 and CCL25 in Foxn1-negative epithelial cells in the post-natal thymus. Int Immunol. 2007;19(2):127–32. . 17158094 10.1093/intimm/dxl129

[18] Ki S, Park D, Selden HJ, Seita J, Chung H, Kim J, et al Global transcriptional profiling reveals distinct functions of thymic stromal subsets and age-related changes during thymic involution. Cell Reports. 2014;9(1):402–15. 10.1016/j.celrep.2014.08.070 25284794 PMC4194175

[19] Ashfield R, Patel AJ, Bossone SA, Brown H, Campbell RD, Marcu KB, et al MAZ-dependent termination between closely spaced human complement genes. EMBO J. 1994;13(23):5656–67. . 7988563 10.1002/j.1460-2075.1994.tb06904.x PMC395531

[20] Rode I, Martins VC, Kublbeck G, Maltry N, Tessmer C, Rodewald HR. Foxn1 Protein Expression in the Developing, Aging, and Regenerating Thymus. J Immunol. 2015;195(12):5678–87. 10.4049/jimmunol.1502010 . 26538393

[21] Ucar A, Ucar O, Klug P, Matt S, Brunk F, Hofmann TG, et al Adult Thymus Contains FoxN1(-) Epithelial Stem Cells that Are Bipotent for Medullary and Cortical Thymic Epithelial Lineages. Immunity. 2014;41(2):257–69. Epub 2014/08/26. 10.1016/j.immuni.2014.07.005 25148026 PMC4148705

[22] Muzumdar MD, Tasic B, Miyamichi K, Li L, Luo L. A global double-fluorescent Cre reporter mouse. Genesis. 2007;45(9):593–605. 10.1002/dvg.20335 . 17868096

[23] Gray D, Abramson J, Benoist C, Mathis D. Proliferative arrest and rapid turnover of thymic epithelial cells expressing Aire. J Exp Med. 2007;204(11):2521–8. . 17908938 10.1084/jem.20070795 PMC2118482

[24] Sekai M, Hamazaki Y, Minato N. Medullary thymic epithelial stem cells maintain a functional thymus to ensure lifelong central T cell tolerance. Immunity. 2014;41(5):753–61. 10.1016/j.immuni.2014.10.011 . 25464854

[25] Wong K, Lister NL, Barsanti M, Lim JM, Hammett MV, Khong DM, et al Multilineage potential and self-renewal define an epithelial progenitor cell population in the adult thymus. Cell Reports. 2014;8(4):1198–209. Epub 2014/08/19. 10.1016/j.celrep.2014.07.029 . 25131206

[26] Metzger TC, Khan IS, Gardner JM, Mouchess ML, Johannes KP, Krawisz AK, et al Lineage tracing and cell ablation identify a post-Aire-expressing thymic epithelial cell population. Cell Reports. 2013;5(1):166–79. 10.1016/j.celrep.2013.08.038 24095736 PMC3820422

[27] Wang X, Laan M, Bichele R, Kisand K, Scott HS, Peterson P. Post-Aire maturation of thymic medullary epithelial cells involves selective expression of keratinocyte-specific autoantigens. Front Immunol. 2012;3(March):19 10.3389/fimmu.2012.00019 22448160 PMC3310317

[28] Jenkinson WE, Bacon A, White AJ, Anderson G, Jenkinson EJ. An epithelial progenitor pool regulates thymus growth. J Immunol. 2008;181(9):6101–8. . 18941199 10.4049/jimmunol.181.9.6101

[29] Velardi E, Tsai JJ, Holland AM, Wertheimer T, Yu VW, Zakrzewski JL, et al Sex steroid blockade enhances thymopoiesis by modulating Notch signaling. J Exp Med. 2014;211(12):2341–9. 10.1084/jem.20131289 25332287 PMC4235646

