# Supplementary material for: Foxn1 Is Dynamically Regulated in Thymic Epithelial Cells during Embryogenesis and at the Onset of Thymic Involution
Source: PLoS One. 2016 Mar 16;11(3):e0151666. doi: 10.1371/journal.pone.0151666 (PMC4794177; doi:10.1371/journal.pone.0151666)
Supplement: S1 Fig — (PDF) [file pone.0151666.s001.pdf]

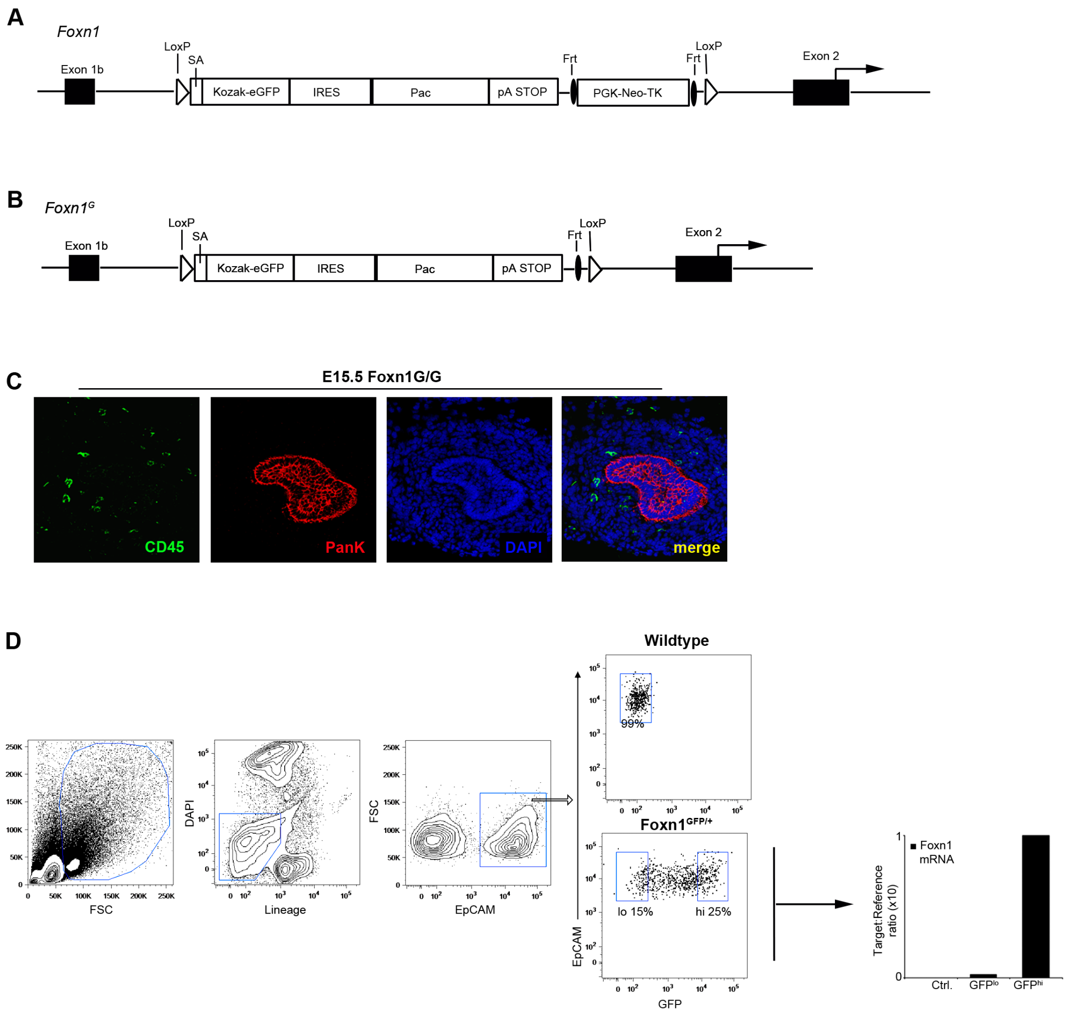

S1 Fig. Characterization of the Foxn1^{GFP} allele.

(A) A LoxP-flanked splice acceptor (SA)-eGFP-IRES-Pac-polyA-CMAZ transcriptional pause (Stop) cassette encompassing a Frt-flanked PGK-neomycin resistance -TK cassette, was inserted into intron 1b of the *Foxn1* genomic locus by homologous recombination in sv129/ola ES cells (line E14tg2a) ES cells, using homology arms previously used to generate the *Foxn1^{R}* allele[1]. G418 resistant colonies were picked and correct targeting was verified by Southern blotting. Two independent correctly targeted ES cell lines were used to generate chimeric mice. Germline transmission was verified by coat color and Southern blotting, and the chimeric founders were used to generate lines of transgenic mice that carried the targeted allele. *Pac* encodes puromycin resistance. The eGFP cDNA contained an optimized Kozak sequence. The *Foxn1* translational start is in exon 2. **(B)** The Frt –flanked selectable marker cassette was removed from the targeted allele by breeding the transgenic founders with mice that constitutively expressed Flpe recombinase, to generate the *Foxn1^{G}* reporter strain. Mice from this strain were subsequently backcrossed onto the C57BL/6 background for at least 10 generations. (**C**) *Foxn1^{G}* is a functional null allele, as demonstrated by failure of the *Foxn1$^{G/G}$* thymic rudiment to undergo colonization by hematopoietic cells (CD45^{+} cells) at any stage in ontogeny, shown for E15.5 embryonic thymi. Image shows cryosection from an E15.5 *Foxn1$^{G/G}$* mouse embryo after staining with the markers shown. PanK (pancytokeratin) identifies the thymic primordium. No CD45^{+} cells are present within the *Foxn1$^{G/G}$* thymic rudiment. Image is representative of more than 3 independent analyses. **(D)** eGFP faithfully reports *Foxn1* mRNA expression. GFP^{lo} and GFP^{hi} EpCAM^{+} TECs were isolated from adult Foxn1$^{GFP/+}$ mice by flow cytometry as shown and 100 cells from each population was processed for analysis by RT-qPCR as shown (right hand panel). (**C,D**)**,** n is greater than 3 independent biological analyses.
