# Supplementary material for: Foxn1 Is Dynamically Regulated in Thymic Epithelial Cells during Embryogenesis and at the Onset of Thymic Involution
Source: PLoS One. 2016 Mar 16;11(3):e0151666. doi: 10.1371/journal.pone.0151666 (PMC4794177; doi:10.1371/journal.pone.0151666)
Supplement: S1 Table — (PDF) [file pone.0151666.s002.pdf]

S1 Table: Antibodies used for flow cytometry and immunohistochemistry

Name	Fluoro-chrome	Host	Mono/ Polyclonal	Antigen	Clone	Manufacturer	Dilution	Valid-ation
EpCAM	APC-Cy7	Rat	Mono	TE-71 TEC line	G8.8	Biolegend	1:200	1
EpCAM	PE	Rat	Mono	As above	G8.8	Biolegend	1:800	1
MHCII	PE-Cy7	Rat	Mono	C57/BL6 spleen cells	M5/114.15.2	Biolegend	1:1000	1
MHCII	APC-eFluor780	Rat	Mono	As above	M5/114.15.2	eBioscience	1:800	1
UEA1	Biotin				Cat. No. B-1065	Vector Labs	1:800	1
Ly-51	PE	Rat	Mono	Lymphoma cell line, T-cell clones	6C3	Biolegend	1:800	1
CD205	PE-Cy7	Rat	Mono		205yekta	eBioscience	1:40	2
TCRβ	PerCP-Cy5.5	Hamster	Poly	Purified TCR	H57-597	Biolegend	1:400	1
CD4	PerCP-Cy5.5	Rat	Mono		RM4-5	eBioscience	1:400	1
CD8	PerCP-Cy5.5	Rat	Mono		53-6.7	eBioscience	1:400	1
Ter119	PerCP-Cy5.5	Rat	Mono		Ter119	eBioscience	1:400	1
Cd11c	PerCP-Cy5.5	Hamster	Poly		N418	eBioscience	1:400	1
CD31	PerCP-Cy5.5	Rat	Mono	Haematopoietic cell line	390	Biolegend	1:400	1
Ki67	PE	Rat	Mono		SolA15	eBioscience	1:400	3
Active Caspase-3	PE	Rabbit	Poly	Human active caspase-3	C92-605	BD Biosciences	1:5	4
Streptavidin	BV650				Cat. No. 405231	Biolegend	1:500	

1. Bredenkamp *et al*. 2014. Development 141: 1627-1637.
2. Baik *et al*. 2013. European Journal of Immunology 43: 589–594.
3. Cowan *et al.* 2014. Journal of Immunology 193: 1204–1212.
4. Small and Kraal 2003. International Immunology 15: 197-203.
