# Supplementary material for: Foxn1 Is Dynamically Regulated in Thymic Epithelial Cells during Embryogenesis and at the Onset of Thymic Involution
Source: PLoS One. 2016 Mar 16;11(3):e0151666. doi: 10.1371/journal.pone.0151666 (PMC4794177; doi:10.1371/journal.pone.0151666)
Supplement: S2 Table — (PDF) [file pone.0151666.s003.pdf]

S2 Table: Primers used for RT-qPCR

Gene	Left primer (5’ to 3’)	Right primer (5’ to 3’)
Aire	GGTTCCTCCCCTTCCATC	GGCACACTCATCCTCGTTCT
Ccl25	GAGTGCCACCCTAGGTCATC	CCAGCTGGTGCTTACTCTGA
Ccrl1	CCTCTCCCAGCTTAAACAGC	AGTATTCCGCTGACTGGTTCA
Cd40	CCATGTGACTCAGGCGAAT	TAACCCGAAGCCCTTGATT
Cd80	TCGTCTTTCACAAGTGTCTTCAG	TTGCCAGTAGATTCGGTCTTC
Dll4	AGGTGCCACTTCGGTTACAC	GGGAGAGCAAATGGCTGATA
Foxn1	TGACGGAGCACTTCCCTTAC	GACAGGTTATGGCGAACAGAA
Hmbs	TCCCTGAAGGATGTGCCTAC	AAGGGTTTTCCCGTTTGC
Hprt	TCCTCCTCAGACCGCTTTT	CCTGGTTCATCATCGCTAATC
Kitl	GCGCTGCCTTTCCTTATG	TCCTTGGTTTTGACAAGAGGAT
Ywhaz	CTTCCTGCAGCCAGAAGC	GGGTTTCCTCCAATCACTAGC
